# Mass spectrometry reveals the presence of specific set of epigenetic DNA modifications in the Norway spruce genome

**DOI:** 10.1038/s41598-019-55826-z

**Published:** 2019-12-17

**Authors:** Igor A. Yakovlev, Daniel Gackowski, Abdulkadir Abakir, Marcos Viejo, Alexey Ruzov, Ryszard Olinski, Marta Starczak, Carl Gunnar Fossdal, Konstantin V. Krutovsky

**Affiliations:** 1 Norwegian Institute for Bioeconomy Research, Pb 115, NO-1431, Ås, Norway; 2 Department of Clinical Biochemistry, Collegium Medicum, Nicolaus Copernicus University, ul. Karlowicza 24, PO-85-092 Bydgoszcz, Poland; 3 Wolfson Centre for Stem Cells, Tissue Engineering and Modelling (STEM), Division of Cancer and Stem Cells, School of Medicine, Centre for Biomolecular Sciences, University of Nottingham, University Park, Nottingham, NG7 2RD UK; 4 Department of Forest Genetics and Forest Tree Breeding, Georg-August University of Göttingen, Büsgenweg 2, Göttingen, 37077 Germany; 5 Laboratory of Population Genetics, N. I. Vavilov Institute of General Genetics, Russian Academy of Sciences, Moscow, 119991 Gubkina Str. 3, Russian Federation; 6 Laboratory of Forest Genomics, Genome Research and Education Center, Institute of Fundamental Biology and Biotechnology, Siberian Federal University, Akademgorodok 50a/2, Krasnoyarsk, 660036 Russian Federation; 7 Department of Ecosystem Science and Management, Texas A&M University, College Station, Texas 77843-2138 USA

**Keywords:** DNA methylation, Epigenomics

## Abstract

5-Methylcytosine (5mC) is an epigenetic modification involved in regulation of gene expression in metazoans and plants. Iron-(II)/α-ketoglutarate-dependent dioxygenases can oxidize 5mC to 5-hydroxymethylcytosine (5hmC), 5-formylcytosine (5fC) and 5-carboxylcytosine (5caC). Although these oxidized forms of 5mC may serve as demethylation intermediates or contribute to transcriptional regulation in animals and fungi, experimental evidence for their presence in plant genomes is ambiguous. Here, employing reversed-phase HPLC coupled with sensitive mass spectrometry, we demonstrated that, unlike 5caC, both 5hmC and 5fC are detectable in non-negligible quantities in the DNA of a conifer, Norway spruce. Remarkably, whereas 5hmC content of spruce DNA is approximately 100-fold lower relative to human colorectal carcinoma cells, the levels of both - 5fC and a thymine base modification, 5-hydroxymethyluracil, are comparable in these systems. We confirmed the presence of modified DNA bases by immunohistochemistry in Norway spruce buds based on peroxidase-conjugated antibodies and tyramide signal amplification. Our results reveal the presence of specific range of noncanonical DNA bases in conifer genomes implying potential roles for these modifications in plant development and homeostasis.

## Introduction

Epigenetic mechanisms are instrumental in plant development and adaptation to environmental stress^1,2^. DNA methylation (5-methylcytosine, 5mC) is a conserved epigenetic mark that is associated with transcriptional regulation in both plants and animals^3–5^. The patterns of DNA methylation dynamically change during cellular differentiation contributing to tissue- and developmental stage-specific gene expression in these organisms^3^. In metazoans, fungi and algae, 5mC can be oxidized to 5-hydroxymethylcytosine (5hmC), 5-formylcytosine (5fC) and 5-carboxylcytosine (5caC) by the members of the TET/JBP (ten-eleven translocation/J-binding proteins) family of iron-(II)/α-ketoglutarate-dependent dioxygenases^6–10^. Since both 5fC and 5caC can be recognized and removed from DNA by thymine-DNA glycosylase (TDG) followed by subsequent regeneration of the abasic site with unmodified cytosine via the base excision repair (BER) pathway, these modifications may serve as intermediates in active DNA demethylation in mammals^11^. Moreover, as the maintenance DNA methyltransferase, DNMT1, prefers hemi-methylated (5mC/C) substrates over hemi-hydroxymethylated (5hmC/C) ones, 5hmC may be instrumental in passive replication-dependent demethylation of DNA in vertebrates^12^. In contrast with animal systems, in plants, 5mC can be directly recognized and excised from DNA by specific DNA glycosylases, such as ROS1 and DME, without the preceding enzymatic oxidation^3,13^. In line with these differences in the mechanisms of DNA demethylation, TET/JBP dioxygenases have not been identified in plants to date^14^, and experimental evidence supporting the presence of the oxidized forms of 5mC (referred together as oxi-mCs) in the plant genomes is very limited and ambiguous^15–18^. Thus, although an early study based on chemical derivatization coupled with liquid chromatography/tandem mass spectrometry reported the presence of all three oxi-mCs in a number of plant species^17^, and, according to another study, 5hmC was found to be localized in transcriptionally silent transposable elements in rice^18^, there are conflicting reports on the prevalence of this DNA modification in *Arabidopsis*^15–17^. In the present study, we aimed to systematically examine the levels of non-canonical DNA bases in the buds of Norway spruce (*Picea abies*), a coniferous tree that is extensively studied in long–term breeding programs and is often regarded as one of the “model” species for plant epigenetics^19,20^. In addition, we wanted to compare the abundance of these DNA modifications in spruce with those of human colorectal carcinoma (HCT 116) and human embryonic stem cells (hESCs), which are well-studied regarding DNA modifications^21^.

## Results and Discussion

To determine the prevalence of 5mC, its oxidized derivatives as well as a thymine base modification, 5-hydroxymethyluracil (5hmU)^22^ together with the products of DNA base damage (deoxyuridine and 8-oxo-2′-deoxyguanosine)^23–25^ in our samples, we employed a sensitive two dimensional ultra-performance liquid chromatography tandem mass-spectrometry (2D-UPLC–MS/MS) that we previously successfully used for quantification of these DNA modifications in cancer cell lines and tumor tissue^26,27^ (Supplementary Fig. S1 and Table S1). In addition, we confirmed the presence of modified DNA bases by immunohistochemistry in Norway spruce buds based on peroxidase-conjugated antibodies and tyramide signal amplification.

**Figure 1:**
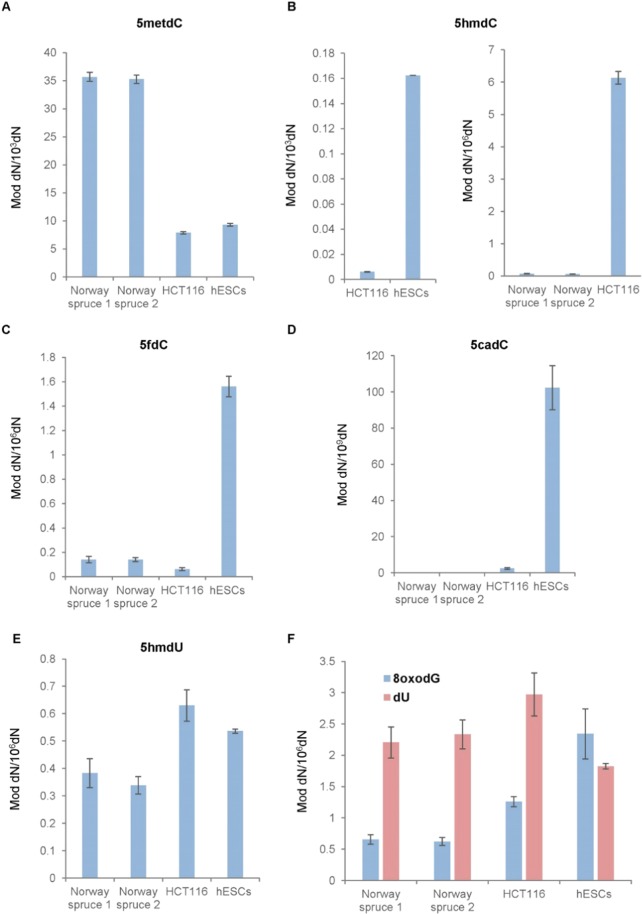
Mass-spectrometry (MS) quantification of noncanonical bases in the DNA isolated from the Norway spruce bud tissues 1 and 2 and from human colorectal carcinoma (HCT 116) and embryonic stem cells (hESCs): (**A)** 5-methyl-2′-deoxycytidine, (**B**) 5-(hydroxymethyl)-2′-deoxycytidine, (**C**) 5-formyl-2′-deoxycytidine, (**D**) 5-carboxy-2′-deoxycytidine, (**E**) 5-(hydroxymethyl)-2′-deoxyuridine, (**F**) 2′-deoxyuridine and 8-oxo-2′-deoxyguanosine. Vertical line across the top of the bars represents ± standard deviation.

**Figure 2:**
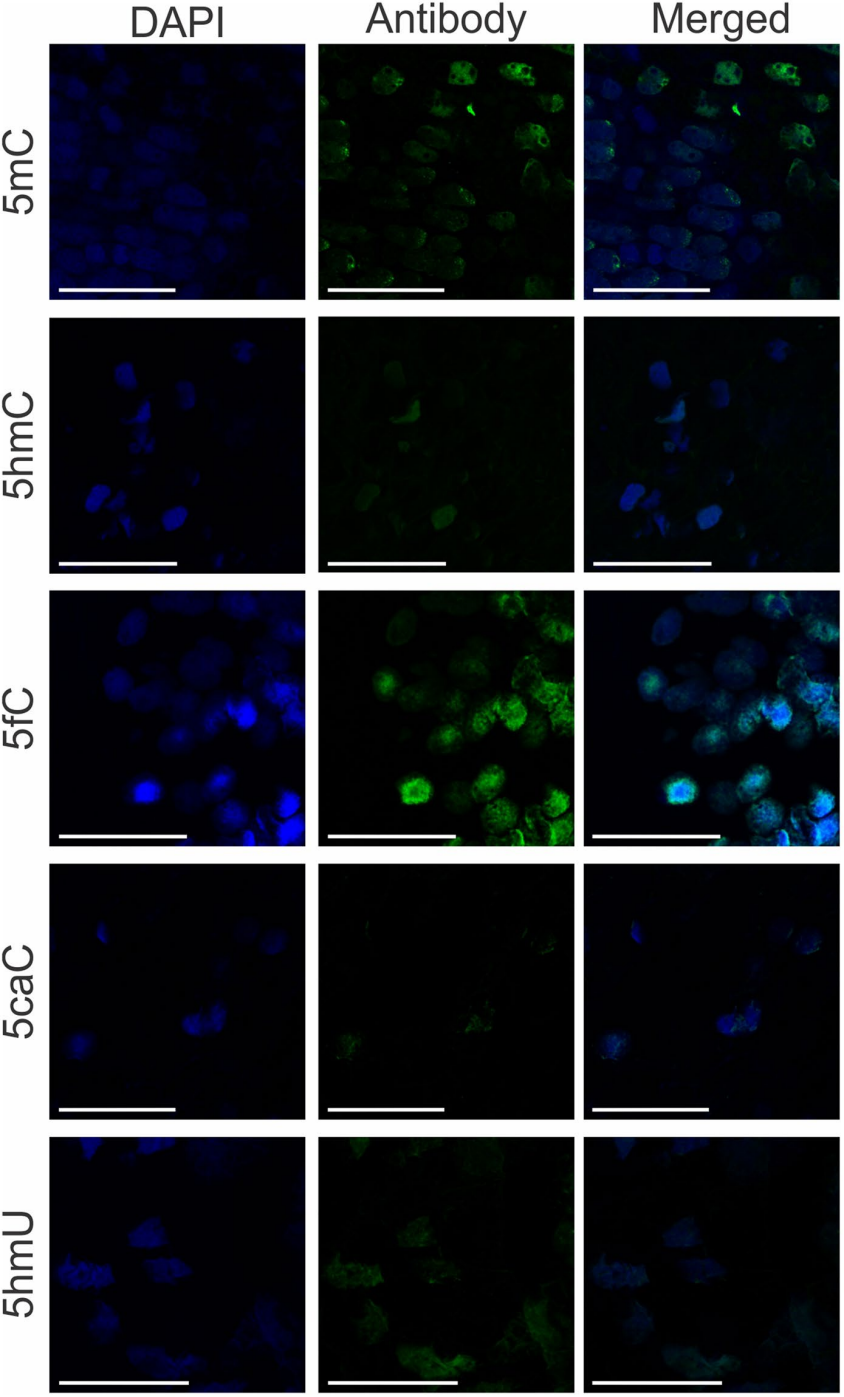
Immunodetection of DNA modified bases in the Norway spruce bud tissue. “DAPI” demonstrates nuclei position, “Antibody” - the modified base fluorescence, and “Merged” - the overlapping of “DAPI” and “Antibody” signals. Antibodies signal coincides with the nucleus as expected. 5mC and 5fC display the highest fluorescence, whereas 5hmU and 5hmC are much lower, and 5caC is the lowest. Microscope magnification is 63 × . Bars are 50 µm. Negative controls for DNA modified bases immunodetection shown in Fig. S3.

We did not find any significant difference in general modification levels between epitypes. In agreement with previous studies reporting elevated levels of cytosine methylation in plants^28^, we found that global 5mC content of the spruce DNA was considerably (3.5–4.5 fold) higher compared with human cells (Fig. 1A). Interestingly, previously published genome-wide bisulfite sequencing profiling of Norway spruce also revealed very high levels of 5mC in CG and CHG sequence contexts across the whole genome (74.7% and 69.1% of all the cytosine residues in the corresponding contexts, respectively)^29^, as well as relatively high levels of DNA methylation in the coding regions (21% in CG, 11% in CHG and 1.3% in CHH contexts)^30^. Remarkably, our analyses demonstrated that, 5hmC, albeit detectable in the spruce DNA, was present in the plant samples at approximately 100-fold lower level relative to human colorectal carcinoma cells that are, present in most of the cancers, characterized by global loss of this modification compared to normal mammalian tissue^31^ (Fig. 1B). Accordingly, we detected fluorescence signal for both 5mC and 5hmC in the bud tissues (Fig. 2). Unlike 5hmC, the 5fdC content of Norway spruce buds was comparable with that of HCT 116 cells (Fig. 1C), whereas 5caC was undetectable in the all examined conifer samples (Fig. 1D). Correspondingly, both 5fC and 5caC were immunodetected in the bud tissues (Fig. 2), contradicting the mass spectrometry results regarding 5caC and suggesting that the content of these variants might be development-dependent since there was one-month difference between collection dates for both techniques. Moreover, the levels of 5hmU in the DNA derived from Norway spruce and from the human cell lines were also similar (Fig. 1E), and it was also possible to immunodetect it in the tissues (Fig. 2). A recent observation demonstrated that 5hmU and 5fC are recognized by specific proteins^32^ and that 5fC is rich in active enhancers involved in tissue development/differentiation^33^.

Importantly, analogous to the previously published results^34,35^, none of the oxidized derivatives of 5mC correlated with the levels of the products of DNA damage (deoxyuridine and 8-oxo-2′-deoxyguanosine) in the spruce samples (Fig. 1F) implying that spontaneous free radical-dependent generation of 5hmC and 5fC in plant DNA is extremely unlikely. Although we were unable to identify TET/JBP homologues in Norway spruce using currently available gene models^36^, the specific pattern of oxidized forms of 5mC that we observed in the spruce DNA (near absent 5hmC paralleled by relatively high 5fC and undetectable 5caC) also suggests probable enzymatic origin of these modifications in conifers. In this context, it is worth noting that fungal TET homologue from *Coprinopsis cinerea* exhibited a preference to producing 5fC over other oxi-mCs^9^. Furthermore, a recent report on TET-mediated epimutagenesis of *Arabidopsis* methylome implies the existence of efficient enzymatic machinery allowing removal of 5hmC from DNA and, thus, effectively 5hmC-dependent demethylation, in plants^37^. Correspondingly, as 5hmU is produced via TET/JBP-mediated oxidation of thymine in both kinetoplastids^38^ and also, likely, in mammalian cells^39^, our results may indicate both enzymatic origin and potential biological function of this DNA modification in Norway spruce.

## Conclusions

Collectively, our data reveal and confirm the presence of specific set of modified DNA bases in the spruce genome implying their probable non-spontaneous generation in conifers. It is also possible that these epigenetic modifications may play some role to sense environmental changes and cope with the harsh conditions the spruce trees have to pass through. Therefore, further studies are warranted to understand potential roles of these modifications in plant development and homeostasis.

## Materials and Methods

### Plant material and DNA extraction

DNA samples were collected from the two different epitypes of Norway spruce at the experimental plot in Hoxmark (Norway) in late June after growth cessation and bud formation. Buds were collected from 13-year-old Norway spruce trees produced *in vitro* at two culturing conditions (18 °C – cold epitype (1) and 28 °C – warm epitype (2) from somatic embryos obtained from a single seed originated in a controlled cross of defined parents (♀#2650 × ♂#2707) of Norway spruce performed in outdoor conditions, as previously described^40^. Genomic DNA was isolated from terminal and lateral buds of individual trees of Norway spruce using DNeasy Plant Mini Kit (#69104, Qiagen, UK) according to the manufacturer’s instructions in several rounds in order to obtain around 50 μg of total DNA. The samples were pooled, precipitated with ethanol and dissolved in deionized water.

### Cell culture

HCT 116 cells were maintained on DMEM (GIBCO) supplemented with 10% bovine serum. HUES7 hESCs were cultured in Essential 8™ (E8) medium with supplement (#A1517001) on Matrigel™-coated tissue culture flasks at 37 °C with 5% CO_2_. Cells were passaged every 3–4 d using TrypLE™ Select Enzyme (#12563029). Genomic DNA from cell cultures was isolated according to standard procedures.

### Mass spectrometry

DNA samples were incubated with 1 U of nuclease P1 (Sigma-Aldrich) and tetrahydrouridine (Calbiochem) (cytidine deaminase inhibitor, 10 μg per sample) for 1 h at 37 °C followed by addition of 12 μl of 5% (v/v) NH_4_OH (JT Baker) and 1.3 U of alkaline phosphatase (Sigma-Aldrich) and additional 1 h incubation at 37 °C. The DNA hydrolysates were acidified with CH_3_COOH (Sigma-Aldrich) to final v/v concentration of 2% and ultrafiltered prior to injection. The 2D-UPLC–MS/MS analyses were performed according to the method described earlier by Gackowski *et al*.^27^ with some modifications.

Briefly, DNA hydrolysates were spiked with a mixture of internal standards in volumetric ratio 4:1, to concentration of 50 fmol/µL of [D_3_]-5-(hydroxymethyl)-2′-deoxycytidine, [^13^C_10_, ^15^N_2_]-5-formyl-2′-deoxycytidine, [^13^C_10_, ^15^N_2_]-5-carboxy-2′-deoxycytidine, and [^13^C_10_, ^15^N_2_]-5-(hydroxymethyl)-2′-deoxycytidine, [^13^C,^15^N_2_]-deoxyuridine and [^15^N_5_]-8-oxo-2′-deoxyguanosine. Chromatographic separation was performed with a Waters Acquity 2D-UPLC system with photo-diode array detector for the first dimension chromatography (used for quantification of unmodified deoxynucleosides and 5-methyl-2′-deoxycytosine) and Xevo TQ-S tandem quadrupole mass spectrometer (used for second dimension chromatography and compounds analyzed in positive mode after first dimension: 5-(hydroxymethyl)-2′-deoxycytidine and 8-oxo-2′-deoxyguanosine, to assure better ionization at higher acetic acid concentration). At-column-dilution technique was used between first and second dimension for improving retention at trap/transfer column. The columns used were: a Waters Cortecs T3 column (150 mm × 3 mm, 1.6 µm) with precolumn at the first dimension, a Waters X-select C18 CSH (100 mm × 2.1 mm, 1.7 µm) at the second dimension and Waters X-select C18 CSH (20 mm × 3 mm, 3.5 µm) as trap/transfer column. Chromatographic system operated in heart-cutting mode, indicating that selected parts of effluent from the first dimension were directed to trap/transfer column *via* 6-port valve switching, which served as “injector” for the second dimension chromatography system. The flow rate at the first dimension was 0.5 mL/min and the injection volume was 2 µL. The separation was performed with a gradient elution for 10 min using a mobile phase 0.05% acetate (A) and acetonitrile (B) (0.7-5% B for 5 min, column washing with 30% acetonitrile and re-equilibration with 99% A for 3.6 min). Flow rate at the second dimension was 0.3 mL/min. The separation was performed with a gradient elution for 10 min using a mobile phase 0.01% acetate (A) and methanol (B) (1-50% B for 4 min, isocratic flow of 50% B for 1.5 min, and re-equilibration with 99% A up to next injection). All samples were analyzed in three to five technical replicates of which technical mean was used for further calculation. Mass spectrometric detection was performed using the Waters Xevo TQ-S tandem quadrupole mass spectrometer, equipped with an electrospray ionization source. Collision-induced dissociation was obtained using argon 6.0 at 3 × 10^−6^ bar pressure as the collision gas. Transition patterns for all the analyzed compounds, as well as specific detector settings were determined using the MassLynx 4.1 Intelli-Start feature in quantitative mode to assure best signal-to noise ratio and resolution of 1 at MS1 and 0.75 at MS2 (Table S2). Calibration curves for MS-detected compounds, recovery, limits of detection and quantitation are presented in Fig. S2. Data were processed in Excel. Means, SDs and RSDs were calculated from 3-5 technical replicates.

### Immunodetection of DNA modifications

The presence of 5mC, 5hmdC, 5fC, 5caC and 5hmU in Norway spruce was assessed by immunohistochemical analysis in dormant apical buds collected in mid-July. We used a Tyramide-based amplification method^41^ to be able to detect the low-abundant DNA modifications in the tissues (5hmC, 5caC, 5fC and 5hmU) while the detection of 5mC did not need of signal amplification, following the below procedure.

Buds were immediately fixed after collection in paraformaldehyde 4% overnight at 4 °C in vacuum. Then, buds were sectioned at 12 µm in a cryotome and placed on glass slides at -20 °C until use. The immunodetection was performed at room temperature unless indicated. Ultrapure water was used to prepare all the solutions. Solutions were removed after each incubation step.

Tissue sections were first covered with phosphate buffer saline (PBS; NaCl 0.137 M, KCl 2.7 mM, Na_2_HPO_4_ 0.01 M, KH_2_PO_4_ 1.8 mM, pH 7.4) for 5 min and then permeabilized with increasing and decreasing ethanol solutions (25%, 50%, 75%, 100% in PBS) for 5 min each. After washing in PBS for 5 min, cell walls were permeabilized for 30 min at 45 °C with 2% cellulase Onozuka R-10 in PBS (w/v), pH 4.5 in a humid chamber. Sections were washed for 5 min in PBS prior to cell membrane permeabilization with PBS containing 0.05% Tween 20 (v/v) for 30 min. Then, they were washed twice in PBS for 10 min each. The antigen retrieval was performed with a proteinase K treatment for 2 min at 55 °C (20 mg/ml proteinase K in 40% glycerol and 10 mM Tris-HCl, 1 mM calcium acetate and pH 7.5). After washing in PBS for 5 min, the DNA was denaturized for 30 min in a 4 N HCl solution. The sections were washed in PBS for 5 min and then incubated in BLOXALL^TM^ for 20 min to inactivate endogenous peroxidases and washed again in PBS for 5 min. Then, they were incubated in a blocking solution to avoid unspecific antibody binding (5% BSA in PBS; w/v) for 30 min and incubated in 50 µl of a 1:200 dilution of the primary antibody (mouse anti-5mC clone 33D3 [cat. no. MABE146; Sigma-Aldrich], goat anti- 5hmC [cat. no. 39092; Active motif], rabbit anti 5fC [cat. no. 61223; Active motif], rabbit anti 5caC [cat. no. 61225; Active motif], mouse anti 5hmdU [cat. no. #ab19735; Abcam, UK] in blocking solution (1% BSA in PBS; w/v) for 1 hour. The excess of antibody was washed twice with PBS containing Tween (as described above) for 10 min each and then were incubated in 50 µl of the corresponding secondary antibody (anti-mouse conjugated with Alexa Fluor 633 (cat. no. A21050, Thermo Fisher) for 5mC, anti-goat horse radish peroxidase (HRP) conjugated (cat. no. A5420, Sigma-Aldrich), anti-rabbit HRP conjugated (cat. no. A0545) in blocking solution (10% BSA in PBS, w/v) for 45 min. Another double wash with Tween 20 was performed (as described above) and sections containing 5hmC, 5caC, 5fC, and 5hmU primary antibodies were incubated in 50 µl of a freshly prepared 1:400 dilution of Tyramide in TSA amplification buffer for 2 min [Tyramide Signal Amplification (TSA) Plus Cyanine 5, cat. no. 415001KT, PerkinElmer]. Immediately after, the sections were washed twice in PBS plus Tween 20 (as previously described) before incubation in a DAPI solution for counteracting staining [1 µg/ml DAPI, 0.1% Tween 20 in water (v/v)] for 15 min. After washing with water 3 times for 5 min each, the samples were let to dry and the mounting medium (cat. no. S3023, Dako) and cover glass were applied.

The samples were visualized in a Leica TCS SP5 running LAS AF software using the appropriate wavelengths for exciting each of the fluorophores. Z-stack function was used to take images in the Z axis. Images were processed with LAS X software from Leica. Negative controls for the secondary antibodies were performed (Fig. S3).

## Supplementary information

Supplementary materials

## Data Availability

The datasets generated and/or evaluated during the current study are available from the corresponding author on reasonable request.
