# Supplementary material for: Mass spectrometry reveals the presence of specific set of epigenetic DNA modifications in the Norway spruce genome
Source: Sci Rep. 2019 Dec 17;9:19314. doi: 10.1038/s41598-019-55826-z (PMC6917789; doi:10.1038/s41598-019-55826-z)
Supplement: Supplementary file 1 — Supplementary materials [file 41598_2019_55826_MOESM1_ESM.docx]

Mass spectrometry reveals the presence of specific set of epigenetic DNA modifications in the Norway spruce genome

Igor A. Yakovlev, Daniel Gackowski, Abdulkadir Abakir, Marcos Viejo, Alexey Ruzov, Ryszard Olinski, Marta Starczak, Carl Gunnar Fossdal, Konstantin V. Krutovsky

**Table S1**. Frequencies of DNA modifications in the genomic DNA of the Norway spruce bud tissue.

| **Genotype** | **Epitype** | **Sample ID** | **dN, nmol/μl** | **%GC** | **5-methyl-2’-deoxycytidine/ 10^3^dN** | **5-(hydroxymethyl)-2’-deoxycytidine/ 10^6^dN** | **5-formyl-2’-deoxycytidine/ 10^6^dN** | **5-carboxy-2’-deoxycytidine/ 10^9^dN** | **2’-deoxy-uridine/**  **10^6^dN** | **5-(hydroxymethyl)-2’-deoxyuridine/ 10^6^dN** | **8-oxo-2’-deoxyguanosine/ 10^6^dN** |
| --- | --- | --- | --- | --- | --- | --- | --- | --- | --- | --- | --- |
| A2K (outdoor) | 18°C - cold | 3790 | 4.43 | 39.9% | 35.24±0.71 | 0.04±0.004 | 0.11±0.004 | nd | 1.94±0.26 | 0.29±0.04 | 0.73±0.03 |
|  |  | 3805 | 4.74 | 39.5% | 36.41±0.75 | 0.06±0.01 | 0.14±0.03 | nd | 2.74±0.22 | 0.28±0.02 | 1.01±0.08 |
|  |  | 3866 | 2.28 | 39.2% | 36.08±0.97 | 0.08±0.01 | 0.15±0.03 | nd | 1.76±0.2 | 0.40±0.05 | 0.82±0.05 |
|  | 28°C - warm | 3811 | 1.90 | 39.5% | 34.97±0.90 | 0.12±0.01 | 0.16±0.03 | nd | 1.78±0.24 | 0.59±0.09 | 1.65±0.05 |
|  |  | 3815 | 4.17 | 39.5% | 36.23±0.83 | 0.07±0.01 | 0.10±0.02 | nd | 2.50±0.36 | 0.26±0.03 | 0.73±0.03 |
|  |  | 3854 | 4.28 | 39.9% | 35.31±0.80 | 0.06±0.01 | 0.13±0.03 | nd | 2.04±0.17 | 0.28±0.03 | 0.92±0.05 |

**Table S2**. Transition patterns, specific detector settings and sources of standards for analyzed compounds.

| **compound name** |  | **ionization mode** | **nominal molecular mass (Da)** | **pseudomolecular ion formulation** | **nominal parent ion (Da)** | **nominal daughter ion (Da)** | **capillary (kV)** | **cone (V)** | **collision (eV)** | **standard source** |
| --- | --- | --- | --- | --- | --- | --- | --- | --- | --- | --- |
| 5-(hydroxymethyl)-2'-deoxycytidine | quantifier | ESI+ | 257 | [M+H]+ | 258 | 124 | 1.2 | 15 | 10 | Berry & Associates, Dexter, MI, USA |
|  | qualifier | ESI+ | 257 | [M+H]+ | 258 | 142 | 1.2 | 15 | 10 |  |
| [D_3_]-5-(hydroxymethyl)-2'-deoxycytidine | quantifier | ESI+ | 260 | [(M+3)+H]+ | 261 | 127 | 1.2 | 15 | 10 | Toronto Research Chemicals, Toronto, Canada |
|  | qualifier | ESI+ | 260 | [(M+3)+H]+ | 261 | 145 | 1.2 | 15 | 10 |  |
| 5-formyl-2'-deoxycytidine | quantifier | ESI- | 255 | [M-H]- | 254 | 121 | 3.5 | 28 | 18 | Berry & Associates, Dexter, MI, USA |
|  | qualifier | ESI- | 255 | [M-H]- | 254 | 138 | 3.5 | 28 | 18 |  |
| [^13^C_10_, ^15^N_2_]-5-formyl-2'-deoxycytidine | quantifier | ESI- | 267 | [(M+12)-H]- | 266 | 128 | 3.5 | 28 | 18 | own synthesis, see M&M section |
|  | qualifier | ESI- | 267 | [(M+12)-H]- | 266 | 145 | 3.5 | 28 | 18 |  |
| 5-carboxy-2'-deoxycytidine | quantifier | ESI- | 271 | [M-H]- | 270 | 110 | 3.5 | 20 | 20 | Berry & Associates, Dexter, MI, USA |
|  | qualifier | ESI- | 271 | [M-H]- | 270 | 93 | 3.5 | 20 | 20 |  |
| [^13^C_10_, ^15^N_2_]-5-carboxy-2'-deoxycytidine | quantifier | ESI- | 283 | [(M+12)-H]- | 282 | 116 | 3.5 | 20 | 20 | own synthesis, see M&M section |
|  | qualifier | ESI- | 283 | [(M+12)-H]- | 282 | 99 | 3.5 | 20 | 20 |  |
| 5-(hydroxymethyl)-2'-deoxyuridine | quantifier | ESI- | 258 | [M-H]- | 257 | 124 | 3.5 | 20 | 15 | Berry & Associates, Dexter, MI, USA |
|  | qualifier | ESI- | 258 | [M-H]- | 257 | 214 | 3.5 | 20 | 10 |  |
| [^13^C_10_, ^15^N_2_]-5-(hydroxymethyl)-2'-deoxyuridine | quantifier | ESI- | 270 | [(M+12)-H]- | 269 | 131 | 3.5 | 20 | 15 | own synthesis, see M&M section |
|  | qualifier | ESI- | 270 | [(M+12)-H]- | 269 | 224 | 3.5 | 20 | 10 |  |
| 2'-deoxyuridine | quantifier | ESI- | 228 | [M-H]- | 227 | 184 | 3.5 | 20 | 12 | Sigma-Aldrich, St. Louis, MO, USA |
|  | qualifier | ESI- | 228 | [M-H]- | 227 | 136 | 3.5 | 20 | 10 |  |
| [^13^C, ^15^N_2_]-2'-deoxyuridine | quantifier | ESI- | 231 | [(M+3)-H]- | 230 | 185 | 3.5 | 20 | 12 | Medical Isotopes, Pelham, NH, USA |
|  | qualifier | ESI- | 231 | [(M+3)-H]- | 230 | 137 | 3.5 | 20 | 10 |  |
| 8-oxo-2'-deoxyguanosine | quantifier | ESI+ | 283 | [M+H]+ | 284 | 168 | 1.2 | 20 | 15 | Sigma-Aldrich, St. Louis, MO, USA |
|  | qualifier | ESI+ | 283 | [M+H]+ | 284 | 140 | 1.2 | 20 | 30 |  |
| [^15^N_5_]-8-oxo-2'-deoxyguanosine | quantifier | ESI+ | 288 | [(M+5)+H]+ | 289 | 173 | 1.2 | 20 | 15 | Cambridge Isotope Laboratories, Tewksbury, MA, USA |
|  | qualifier | ESI+ | 288 | [(M+5)+H]+ | 289 | 145 | 1.2 | 20 | 30 |  |


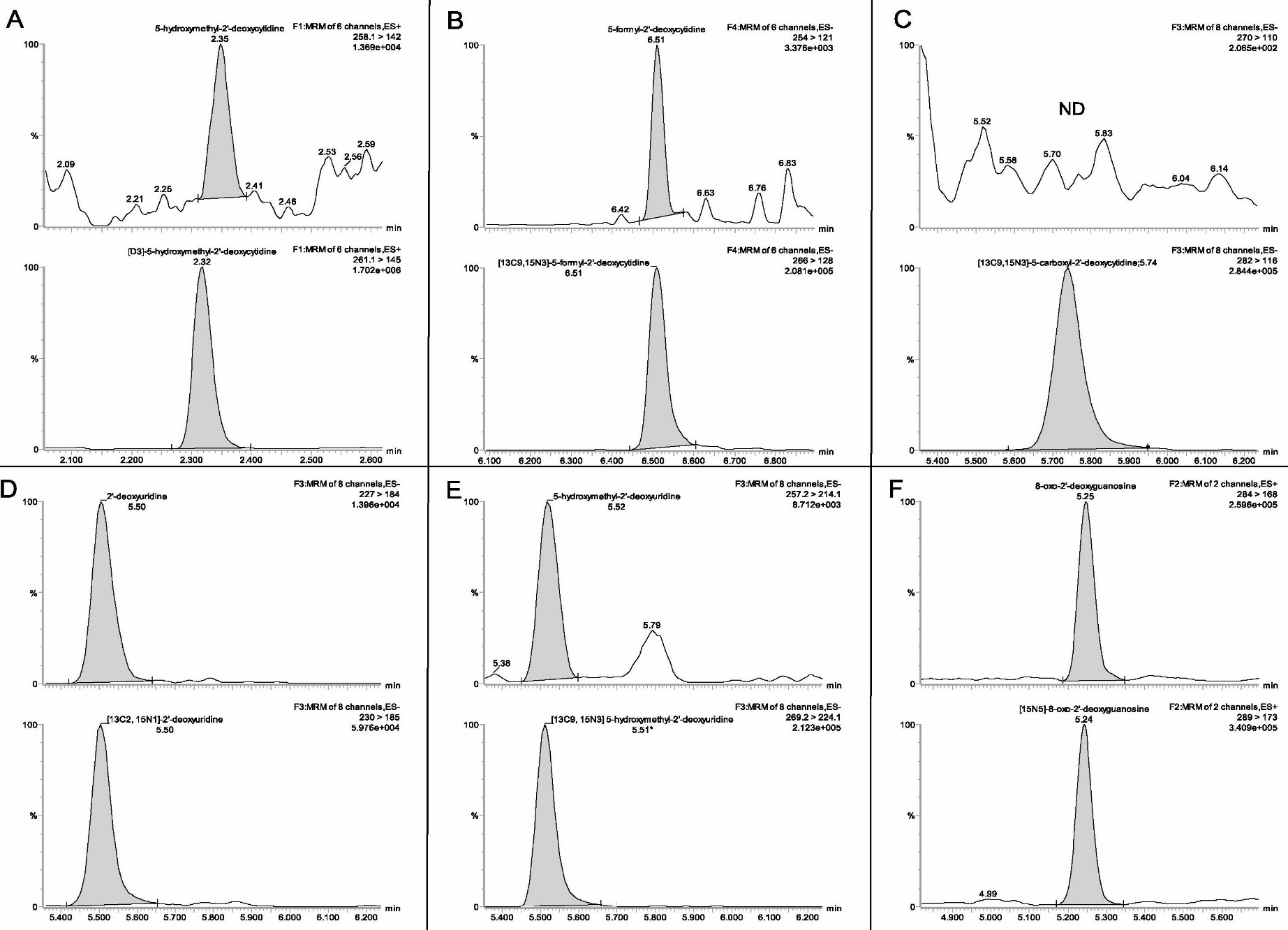


**Figure S1.** The representative UPLC-MS/MS chromatograms of 2 µL of hydrolyzed genomic DNA of the Norway spruce bud tissue (sample ID 3805) spiked with stable-isotope labelled internal standards. (**A**) 5-(hydroxymethyl)-2’-deoxycytidine, (**B**) 5-formyl-2’-deoxycytidine, (**C**) 5- carboxy-2’-deoxycytidine, (**D**) 2’-deoxyuridine, (**E**) 5-(hydroxymethyl)-2’-deoxyuridine, and (**F**) 8-oxo-2’-deoxyguanosine.

**Figure S2.** Calibration curves, recovery, limit of detection (LOD, defined as S/N ratio >3), limit of quantification (LOQ, defined as S/N ratio >10) for (**A**) 5-(hydroxymethyl)-2’-deoxycytidine, (**B**) 5-formyl-2’-deoxycytidine, (**C**) 5-carboxy-2’-deoxycytidine, (**D**) 2’-deoxyuridine, (**E**) 5-(hydroxymethyl)-2’-deoxyuridine, and (**F**) 8-oxo-2’-deoxyguanosine.


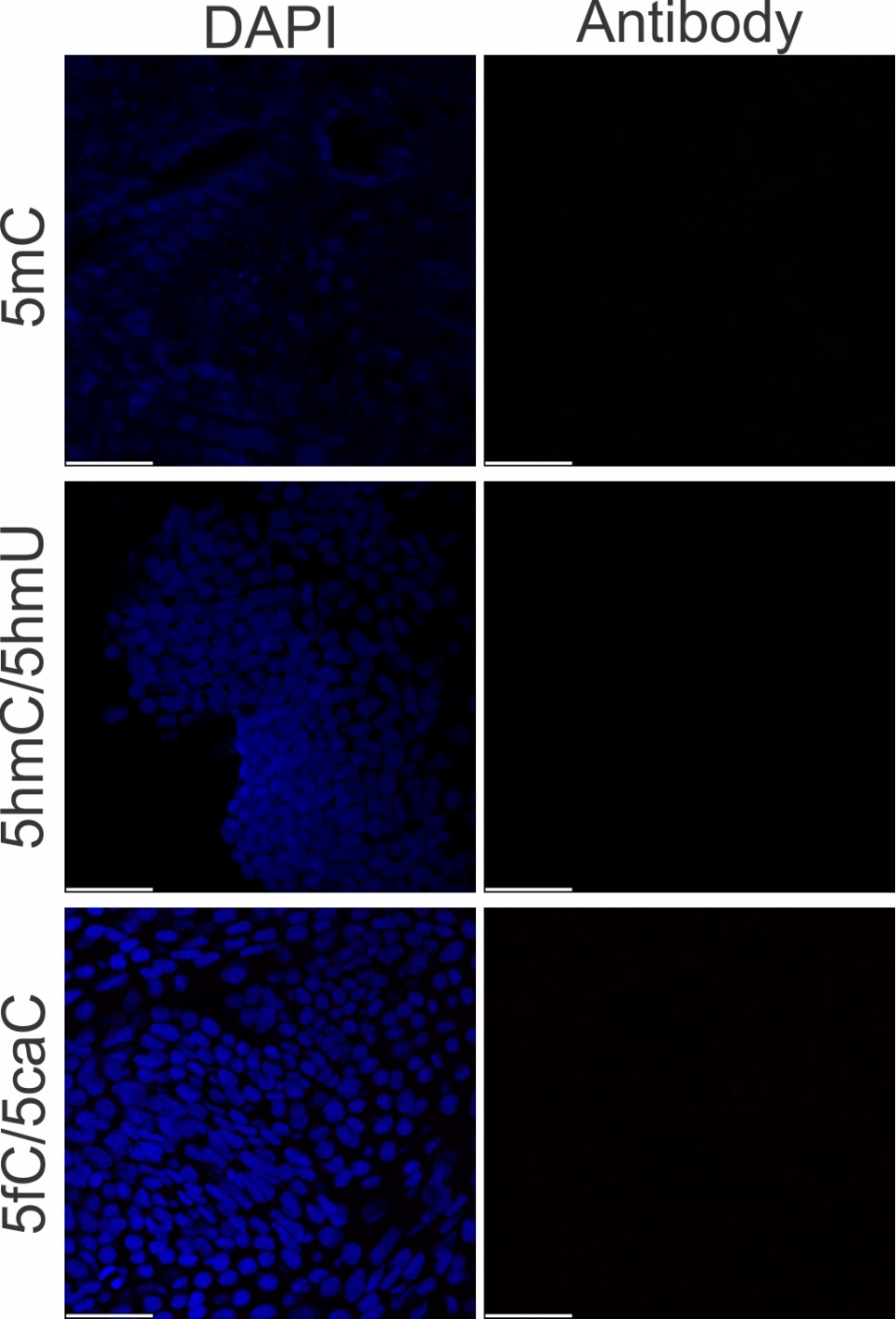


**Figure S3**. Negative controls for the DNA modified bases immunodetection. “DAPI” demonstrates nuclei position and “Antibody” - with the fluorescence generated by unspecific binding of the secondary antibodies (anti-mouse for 5mC, anti-goat for 5hmC/5hmU, and anti-rabbit for 5fC/5caC). Microscope magnification is 63x. Bars are 50 μm.
